# Early Predictors of Objectively Measured Physical Activity and Sedentary Behaviour in 8–10 Year Old Children: The Gateshead Millennium Study

**DOI:** 10.1371/journal.pone.0037975

**Published:** 2012-06-20

**Authors:** Mark S. Pearce, Laura Basterfield, Kay D. Mann, Kathryn N. Parkinson, Ashley J. Adamson, John J. Reilly on behalf of the Gateshead Millennium Study Core Team

**Affiliations:** 1 Institute of Health & Society, Newcastle University, Newcastle upon Tyne, United Kingdom; 2 Human Nutrition Research Centre, Newcastle University, Newcastle upon Tyne, United Kingdom; 3 School of Psychological Sciences and Health, University of Strathclyde, Glasgow, United Kingdom; 4 Queen Elizabeth Hospital, Gateshead NHS Foundation Trust, Gateshead, United Kingdom; 5 Department of Psychology, University of Durham, Durham, United Kingdom; 6 Fleming Nuffield Unit, Child and Adolescent Mental Health Service, Newcastle upon Tyne, United Kingdom; 7 Community Child Health, PEACH Unit, Yorkhill Hospitals, Glasgow, United Kingdom; University of Southampton, United Kingdom

## Abstract

**Background:**

With a number of studies suggesting associations between early life influences and later chronic disease risk, it is suggested that associations between early growth and later physical activity (PA) may be a mediator. However, conflicting evidence exists for association between birth weight and childhood PA. In addition, it is important to know what other, potentially modifiable, factors may influence PA in children given its’ association with childhood and later adiposity. We used the Gateshead Millennium Study (GMS) to identify predictors of childhood PA levels.

**Methods:**

The GMS is a cohort of 1029 infants born in 1999–2000 in Gateshead in northern England. Throughout infancy and early childhood, detailed information was collected. Assessments at age 9 years included body composition, objective measures of habitual PA and a range of lifestyle factors. Mean total volumes of PA (accelerometer count per minute, cpm) and moderate-vigorous intensity PA (MVPA), and the percentage of time spent in sedentary behaviour (%SB) were quantified and related to potential predictors using linear regression and path analysis.

**Results:**

Children aged 8–10 years were included. Significant differences were seen in all three outcome variables between sexes and season of measurement (p<0.001). Restricting children’s access to television was associated with decreased MVPA. Increased paternal age was associated with significant increases in %SB (p = 0.02), but not MVPA or total PA. Increased time spent in out of school sports clubs was significantly associated with decreased %SB (p = 0.02). No significant associations were seen with birth weight.

**Conclusion:**

A range of factors, directly or indirectly, influenced PA and sedentary behaviour. However, associations differed between the different constructs of PA and %SB. Exploring further the sex differences in PA would appear to be useful, as would encouraging children to join out of school sports clubs.

## Introduction

Levels of habitual physical activity and sedentary behaviour in childhood are well established as important to both current and future health of children and adolescents [1]–[4]. While studies have reported associations between childhood physical activity and later cardiovascular and metabolic risk markers for adult disease, other reports also suggest links between increased physical activity levels in children and greater self-esteem [5], [6].

Studies of early growth in relation to physical activity have reported inconsistent findings [7]–[9]. If true associations were to be found between early growth and later physical activity, this could be interpreted as physical activity being a potential mediating factor in the associations between early growth and later risk of chronic disease.

However, in addition to early growth there are a wide range of factors that may influence levels of both physical activity and sedentary behaviour in children, including sex [10]–[13] and season [14]–[17]. Differences in participation in non-school based sporting activities [18] and time spent watching television [19]–[21] and playing electronic games [21] may also be predictors of time spent sedentary or in physical activity.

As increasing levels of physical activity, or decreasing levels of sedentary behaviour, are likely to have both short and long-term benefits, it is crucial that factors that influence physical activity levels are identified and their relative importance and mediating pathways assessed. The most recent follow-up of the Gateshead Millennium Study, a sample socio-economically representative of England at age 8–10 years [22], provided such an opportunity to investigate the early determinants of levels of both physical activity and sedentary behaviour in children. Further, the novel use of path analyses on these data, an approach not taken previously in assessing predictors of sedentary behaviour or physical activity, allowed the assessment of mediating associations and relative contributions using longitudinal (i.e. potential predictor data collected at various times since birth), rather than cross-sectional data.

## Methods

### Study Participants

The Gateshead Millennium Study (GMS) began as a prospective study of 1029 infants and their families recruited shortly after birth between June 1999 and May 2000 in Gateshead, an urban district in north east England. Full details of recruitment and measures taken since birth are reported elsewhere [22]. Briefly, all babies born in pre-specified recruiting weeks between June 1999 and May 2000 to Gateshead-resident mothers were eligible to join the study. For the present study, all families who had not previously opted-out from the cohort were sent a letter and information leaflet inviting them to take part.

### Objective Measurement of Physical Activity and Sedentary Behaviour

Physical activity and sedentary behaviour were measured using the Actigraph GT1M models (Actigraph LLC, Pensacola, Florida, USA). Reviews have concluded repeatedly that the Actigraph measures habitual physical activity and sedentary behaviour in children with high practical utility, reasonably high reliability, high validity relative to criterion measures (energy expenditure and direct observation of movement), and negligible reactivity [23]–[25].

In the present study, the Actigraphs were attached to a waist belt, which parents were asked to put on their child when the child woke, and to remove it before the child went to bed, for a period of seven consecutive days. Parents were also asked to note, in a log sheet, periods when and why the Actigraph was removed. Accelerometry data were reduced manually as described previously [26]. In brief, long periods of consecutive ‘zeros’ in the accelerometry record were checked for entries in the log sheet. These periods were rare and usually corresponded to log-sheet records (e.g. showering) which explained the zeros satisfactorily. Accelerometer records which consisted of at least 3 days were included (days of <6 hours excluded), on the *a priori* grounds that reliability of this amount of Actigraph accelerometry in UK children is adequate [27]. Analyses confirmed that for the data from the present study, reliability was high for total volume of physical activity, moderate-vigorous intensity physical activity (MVPA), and sedentary behaviour for the minimum period of accelerometry [26].

The Actigraph accelerometers in the present study were set to summarize activity data in 15 second sampling intervals (epochs), but data were collapsed to 60 second epochs when summarized to allow use of cut-points in accelerometry output as described below. Objectively measured physical activity was measured using two commonly-used constructs: total volume of physical activity (expressed as the mean counts per minute (cpm) over the duration of accelerometry monitoring [28]) and percentage of time spent in MVPA, as well as percentage of time spent in sedentary behaviour. The epoch chosen does not affect measurements of total volume of activity, but tends to misclassify vigorous intensity physical activity as moderate intensity [25]. Measurement of the amount of time spent in sedentary behaviour is largely unaffected by epoch [25].

In order to express accelerometry output in terms of intensity of activity, it is necessary to apply cut-points to the accelerometry data [25]. A recent review found a moderately large body of high quality and consistent evidence from paediatric validation and calibration studies which suggests that the appropriate cut-point to measure MVPA with the Actigraph lies in the range 3100–3600 cpm [25]. The cut-point of Puyau et al (2002) [29] (3200 cpm) was used to define MVPA. The Actigraph GT1M model has been shown to have a consistent bias of −9% relative to the older Actigraph model [30] and so a +9% correction to GT1M data was made before applying any cut-point to define MVPA and sedentary behaviour. A body of high quality and consistent evidence from paediatric calibration and validation studies suggests that an Actigraph cut-point of around 1100 cpm will measure sedentary behaviour with optimal accuracy [31], [32] across a wide paediatric age range [25]. The cut-point of 1100 cpm was therefore used in the present study to define sedentary behaviour. The season of physical assessment was categorised as ‘winter’ (December to February), ‘summer’ (June to August) and a combined category of ‘spring/autumn’(March to May and September to November).

### Data from Birth and Infancy

Sex, birth weight, standardised for gestational age and sex [33], maternal and paternal age, birth order, and maternal education were recorded at birth. Parents received questionnaires at 6 weeks, and 4, 8, and 12 months, which all included questions regarding whether any breast milk was being given at that age. From this, two breast feeding variables (ever and duration) were derived. Socio-economic status was defined as the ward-level Townsend deprivation score [34] for each study member at the time of their birth. The Townsend deprivation score, derived from 2001 census data (via the link between postcodes and ward identifiers), is a summary measure consisting of the proportion of households in the area without a car, with more than one person per room and that are not owner-occupied and also incorporates the number of men (aged 16–64 years) and women (aged 16–59 years) who were unemployed at the time of the census. The higher the score is, the more deprived the area is assumed to be.

### Anthropometrics and Later Childhood Data

Height and weight were measured by trained researchers according to standard protocols using a Leicester portable height measure and a TANITA TBF 300 MA body fat analyser (both Chasmors, London UK). Body mass index (BMI) was calculated in the usual way (weight (kg)/height (m)^2^). Standardised BMI was expressed as z-scores relative to UK 1990 population reference data [35].

At age 8–10 years, parents were sent a questionnaire on their child’s home environment, which covered restriction and supervision of the child watching television and playing electronic games, and how much time their child spent playing electronic games. Parents were also asked about their own television and electronic games use and how many television sets were in the household. Children completed a questionniare with a researcher which asked about school sports clubs and outside-school sports clubs. Children were asked which club they attended, how frequently they attended, and the duration of each club session.

A favourable ethical opinion for this study was obtained from the Newcastle University Ethics Committee. Written consent was obtained from the parent/main carer of each child, and children provided assent to their participation.

### Statistical Analysis

Relationships between the outcome variables (total physical activity, and percentage time spent in MVPA and in sedentary behaviour) and explanatory variables were estimated by multiple linear regression, as were potential interactions between explanatory variables. Unadjusted coefficients, with corresponding 95% confidence intervals were determined to estimate the total influence (i.e. including both direct and indirect effects) of that variable on each outcome measure. To estimate solely the direct effect of each variable (i.e. not mediated through other factors), an adjusted model was constructed using stepwise regression with a p-value of 0.1 used for retention within a model initially before independent predictors were identified through use of a p-value limit of 0.05.

To estimate indirect pathways (i.e. non-independent predictors, which are mediated through other variables), the adjusted model was reconstructed as a path diagram. Variables that were not in the adjusted model (i.e. that were not independently predictive of physical activity outcomes) were then added to the path diagram, and all paths or correlations with p<0.1 were modelled. Construction of the initial model involving categorical variables used data from a matrix of correlations, derived from person, polychoric, polyserial and tetrachoric correlations. The resulting model was reconstructed using the full data to determine a final model. Model fit was assessed using model chi-square (using the Bollen-Stine bootstrap modification, over 50,000 observations), goodness-of-fit index (GFI), comparative fit index (CFI), and root mean square error of approximation (RMSEA). Adequate fit was defined as a chi-square p-value over 0.05, GFI over 0.95, CFI over 0.95, and RMSEA under 0.05, all of which were satisfied.

In order to allow comparison between variables, and estimate relative importance, standardised beta coefficients (β) were derived for each explanatory variable (where a standardised coefficient is the SD change in the outcome variable elicited by a 1 SD change in the explanatory variable). Parameters were estimated using a random-walk (Metropolis) Markov Chain Monte Carlo (MCMC) algorithm. Assuming diffuse uniform priors, the procedure was run for a burn-in sample of 1,000 observations, and an analysis sample of 100,000 observations. 95% Credible Intervals, CrIs, (analogous to confidence intervals) were obtained from the posterior distribution of each parameter. The final convergence statistic for the model was 1.0001.

Given the possibility that BMI and levels of both physical activity and sedentary behaviour may have a bidirectional relationship [6], [13], all analyses and models were repeated both including and excluding the child’s BMI.

All standard statistical analyses were done using the statistical software package Stata, version 10 (StataCorp, College Station, TX) while path analyses were done using AMOS 17.0 (SPSS Inc, Chicago, IL).

## Results

**Figure 1:**
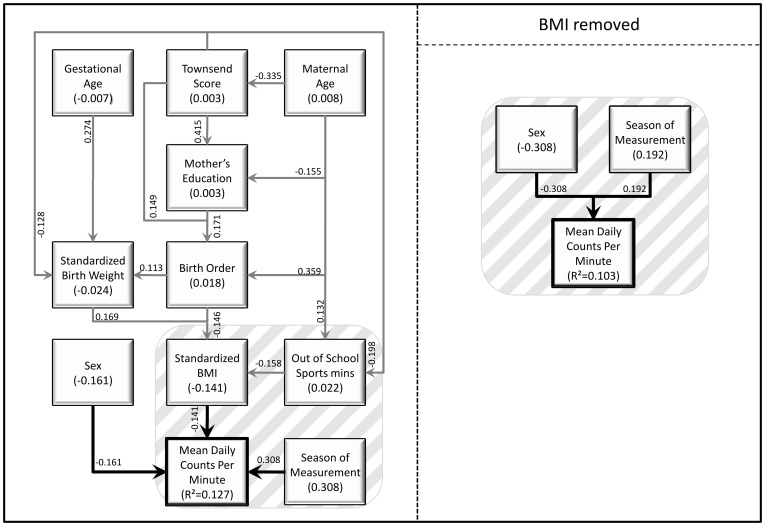
Path diagrams showing the direct and indirect predictors of total PA (mean daily counts per minute), with and without the inclusion of standardised BMI of the child. Significant effects (p<0.05) are represented by solid arrows and are labelled with standardised coefficients (β), with the arrow direction indicating the hypothesised direction of causal flow. Indirect effects are any pathways that are mediated through at least one intermediate (eg, birth order −> BMI −> mean daily counts per minute). Direct effects are represented by arrows going straight from the independent variable to total PA without being mediated through another independent variable. The standardised total effect for each variable is the sum of the direct and indirect effects, and the value is shown underneath the variable name. Error terms and co-variances are omitted for simplicity.

**Figure 2:**
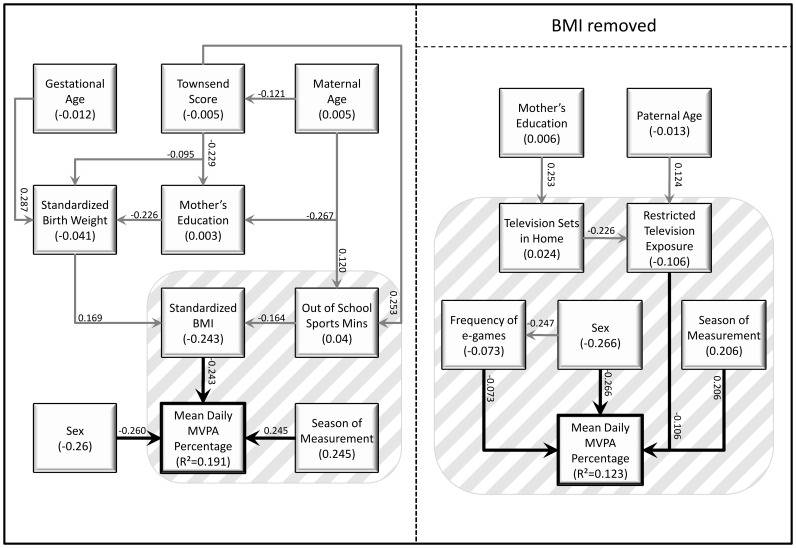
Path diagrams showing the direct and indirect predictors of percent of time spent in MVPA, with and without the inclusion of standardised BMI of the child.

**Figure 3:**
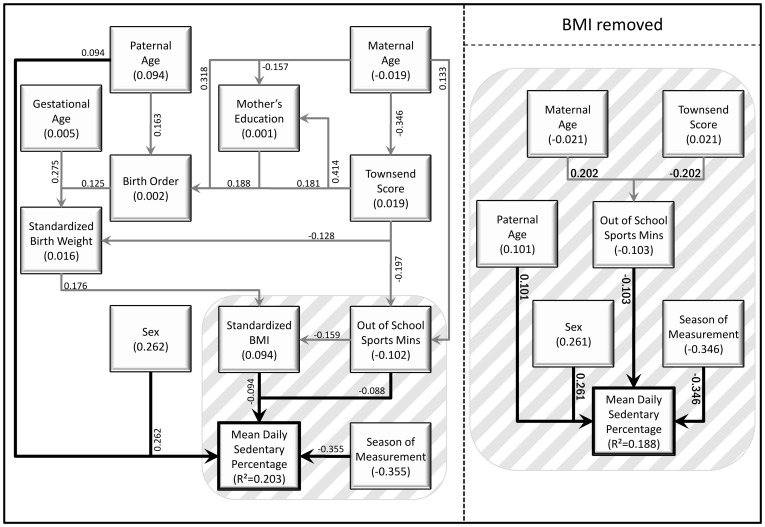
Path diagrams showing the direct and indirect predictors of percent of time spent in sedentary behaviour, with and without the inclusion of standardised BMI of the child.

Of 592 accelerometers given out, 16 were not worn, 24 did not return the corresponding diary, 14 had a software failure, 17 were lost and 1 child was ill throughout the recording period, leaving 520 for potential analysis. Of these, 508 children wore the accelerometer for the required length of time (≥3 days, ≥6 h per day). Twins (n = 26) were excluded from analysis and due to missing data in explanatory variables 143 children had insufficient data for path analysis. There was no significant difference in sexes between the 482 children measured and the 339 children (169 boys and 170 girls) with complete data used in the path analysis (p = 0.192). Descriptive statistics for all continuous variables used in this study, including the physical activity and sedentary behaviour outcome variables, are given in table 1, and for categorical variables in table 2. Table 3 details the significant adjusted associations identified in this study. Figures 1, 2 and 3 illustrate the adjusted models for each outcome variable, with additional indirect associations, in schematic form. Each figure contains two path models, one with the child’s standardised BMI, the other without. The standardised direct effect of each significant relationship is shown, as well as the standardised total effect of each variable (i.e., including both the direct effect and indirect effects mediated through other variables).

**Table 1:** Descriptive statistics, by sex, for continuous variables.

			Male		Female		p-value for sex difference
Variable	n	Mean (SD)	n	Mean (SD)	n	Mean (SD)	
Mean daily counts per minute	482	669.2 (198.4)	231	698.5 (179.0)	251	642.3 (211.4)	0.002
Mean daily MVPA percent	482	4.0 (2.41)	231	4.7 (2.4)	251	3.4 (2.2)	<0.001
Mean daily sedentary percent	482	80.6 (5.3)	231	79.3 (5.4)	251	81.7 (5.0)	<0.001
Standardized Birth Weight	482	−0.1 (1.1)	231	−0.2 (1.1)	251	−0.1 (1.1)	0.921
Mother’s age at child birth (years)	482	28.9 (5.7)	231	29.3 (5.7)	251	28.5 (5.7)	0.083
Father’s age at child birth (years)	465	31.7 (6.3)	227	31.6 (6.1)	238	31.8 (6.6)	0.765
Standardized child BMI year 9	478	0.56 (1.1)	230	0.5 (1.1)	248	0.6 (1.1)	0.537
Gestational age (weeks)	482	39.5 (1.6)	231	39.5 (1.7)	251	39.6 (1.4)	0.411
Total time spent in outof school sports club min per wk	475	62.1 (76.8)	228	67.1 (76.3)	247	57.4 (77.2)	0.267

**Table 2:** Descriptive statistics, by sex, for categorical variables.

		Total		Male		Female	
Variable	Categories	n	%	n	%	n	%
Sex		482	100	231	48	251	52
Townsend quintile	Most Affluent	91	19	53	23	38	15
	2	114	24	45	20	69	28
	3	105	22	52	23	53	21
	4	88	19	39	17	49	20
	Most Deprived	78	16	38	17	40	16
Birth order	1^st^ child	224	47	103	45	121	48
	2^nd^ child	175	36	84	36	91	36
	3^rd^ child	58	12	32	14	26	11
	4^th^ child	15	3	7	3	8	3
	5^th^ child or more	10	2	5	2	5	2
Mother’s qualifications	Degree or equiv	96	21	53	24	43	19
	A level or equiv	50	11	19	9	31	13
	GCSEs or Equiv	228	51	116	53	112	48
	NVQs or equiv	77	17	30	14	47	20
Season of measurement	Winter	120	25	55	24	65	26
	Spring/Autumn	225	47	112	48	113	45
	Summer	137	28	64	28	73	29
How often does the parent restrict the child’s time spent watching TV	Don’t Know	16	4	9	4	7	3
	Never/rarely	88	21	36	18	52	25
	Sometimes	222	54	111	54	111	54
	Often/Very often	86	21	49	24	37	18
How often do you play egames?	Not Very often,	81	17	24	11	57	23
	At least once a week,	56	12	25	11	31	13
	A couple of times a week,	192	41	81	36	111	45
	Every Day,	115	24	82	37	33	13
	Don’t Know	27	6	12	5	15	6
Number of TV sets in home	0 or 1	21	5	11	5	10	5
	2	75	18	38	19	37	18
	3	138	34	72	36	66	32
	4 or more	176	43	82	40	94	45

**Table 3:** Results of adjusted regression analyses showing coefficients for all variables having at least one significant unadjusted association with an outcome variable.

Variable		Co-efficient (95% CI)		p-value
**Mean daily counts per minute**				
Sex	Male	Reference		0.001
	Female	−61.1	(−97.4, −24.7)	
Season of measurement	Winter	Reference		<0.001
	Spr/Aut^1^	117	(72.5, 161.4)	
	Summer	151.9	(102.1, 201.7)	
Standardised BMI		−18.8	(−35.3, −2.4)	0.025
***Excluding BMI***				
Sex	Male	Reference		0.002
	Female	−54.1	(−88.0, −20.2)	
Season of measurement	Winter	Reference		<0.001
	Spr/Aut^1^	115.4	(73.3, 157.5)	
	Summer	136.7	(90.2, 183.2)	
**Mean daily moderate-vigorous intensity physical activity**				
Sex	Male	Reference		
	Female	−1.2	(−1.7, −0.8)	<0.001
Season of measurement	Winter	Reference		<0.001
	Spr/Aut^1^	1.3	(0.7, 2.0)	
	Summer	1.7	(1.0, 2.4)	
Standardised BMI		−0.5	(−0.7, −0.3)	<0.001
***Excluding BMI***				
Sex	Male	Reference		<0.001
	Female	−1.3	(−1.8, −0.8)	
Season of Measurement	Winter	Reference		<0.001
	Spr/Aut^1^	1.2	(0.6, 1.8)	
	Summer	1.3	(0.7, 2.0)	
Restricted TV exposure	Never	Reference		0.0302
	Sometimes	−0.7	(−1.3, −0.2)	
	Often/Very often	−0.4	(−1.1, 0.3)	
**Mean daily sedentary percent**				
Sex	Male	Reference		<0.001
	Female	2.6	(1.6, 3.6)	
Season of measurement	Winter	Reference		<0.001
	Spr/Aut^1^	−3.2	(−4.4, −2.0)	
	Summer	−4.5	(−5.9, −3.2)	
Paternal age		0.1	(0.0, 0.2)	0.05
Time in out of school sports club		−0.01	(−0.01, 0.0)	0.05
Standardised BMI		0.4	(−0.04, −0.01)	0.05
***Excluding BMI***				
Sex	Male	Reference		<0.001
	Female	2.6	(1.6, 3.6)	
Season of measurement	Winter	Reference		<0.001
	Spr/Aut^1^	−3.2	(−4.4, −2.0)	
	Summer	−4.4	(−5.8, −3.1)	
Paternal age		0.1	(0.01, 0.2)	0.035
Time in out of school sports club		−0.01	(−0.01, −0.001)	0.022

1 Spring and autumn.

Study participants included in our analyses were comparable to those that did not provide data during the follow-up at age 8–10 years. No differences were found between those included in analyses and the remaining cohort for sex (p = 0.67), birth weight (p = 0.88), gestational age (p = 0.91) and birth order (p = 0.39). Small differences were seen for maternal and paternal ages with both mother’s and father’s in our analysis group being slightly older (no more than one year difference). The analysis group were also slightly more advantaged in their Townsend socio-economic deprivation scores and maternal qualifications at birth.

### Total PA

The mean daily counts per minute was 669 and was significantly higher in boys than in girls (mean difference 56, 95% CI 21, 91, p = 0.002). In univariate analyses, additional significant associations were seen between mean daily counts per minute and paternal age (b = −3.6 per year, 95% CI −6.4, −0.8, p = 0.01) season of assessment (higher mean daily counts per minute in both summer and spring and autumn than in the winter, p<0.001) and contemporary BMI (b = −24.3 per sd score, 95% CI −40.3, −8.2, p = 0.003). In the adjusted model, only paternal age was no longer significant (Table 3). Excluding BMI from the model made very little difference to the results of the standard regression. However, the path models with and without BMI are very different due to the significant indirect effects of socio-economic deprivation, maternal education, standardised birth weight, birth order, maternal age and time spent in out-of-school sports clubs which were all mediated through contemporary BMI (Figure 1).

### Percent of Time Spent in MVPA

The mean daily percentage of time spent in MVPA was 4.0%, with boys spending significantly greater percentages of time in MVPA than did girls (mean difference 1.2%, 95% CI 0.8, 1.7, p<0.001). At the univariate level, associations were also seen between percentage of time spent in MVPA and paternal age (b = −0.03% per year, 95% CI −0.07, −0.003, p = 0.03), contemporary BMI (b = −0.4% per sd score, 95% CI −0.6, −0.2, p<0.001), season of physical activity assessment (p<0.001), the amount of time spent in all sports clubs (b = 0.002 per minute per week, 95% CI 0.00, 0.01, p = 0.05) and whether or not their exposure to television was restricted by their parents (with decreasing percentage of time spent in MVPA with parental restriction, p = 0.05). In the adjusted model including the child’s standardised BMI, the only other significant adjusted associations were with sex of the child and season of assessment (Table 3). Excluding BMI, sex and season remained significant with the additional significance of parental restrictions on watching television, (the mean daily average percentage of time spent in MVPA was less in those with any level of television restriction).

In addition to the direct effects of season of assessment, sex of the child and restricted television exposure, the path model excluding standardised BMI also included indirect effects of maternal education, paternal age and the number of television sets in the child’s home (Figure 2a). The strongest predictors were sex of the child and season of assessment, followed by parental television restrictions. Including BMI, in addition to the other direct effects of sex of the child and season of assessment, there were indirect effects of Townsend socio-economic deprivation, maternal age and education, standardised birth weight and time spent in out of school sports clubs, all of which acted through their association with standardised BMI (Figure 2b).

### Percent of Time Spent in Sedentary Behaviour

The mean daily percentage of time spent in sedentary behaviour was 80.6%, with boys spending significantly less time in sedentary behaviour than girls (mean difference = 2.5%, 95% CI 1.5, 3.4, p<0.001). At the univariate level, significant associations were also seen between percentage of time spent in sedentary behaviour and paternal age (b = 0.1% per year, 95% CI 0.02, 0.17, p = 0.02), whether the child was ever breast fed (mean difference for yes versus no = 1.2%, 95% CI 0.2, 2.2, p = 0.02), contemporary BMI (b = 0.5% per sd score, 95% CI 0.1, 0.9, p = 0.03), the amount of time spent in all, school and out-of-school sports clubs (b = −0.01% per minute per week, 95% CI −0.01, −0.001, p = 0.02, b = −0.01% per minute per week, 95% CI −0.02, −0.001, p = 0.04 and b = −0.01% per minute per week, 95% CI −0.01, −0.001, p = 0.05 respectively) and the season of assessment (p<0.001). In the adjusted model, being female, having a greater standardised BMI, having an older father and being assessed in the winter were all significantly associated with an increased percentage of time spent in sedentary behaviour, while increased time spent in out of school sports clubs was significantly associated with a decrease in percentage of time spent in sedentary behaviour (Table 3). Similar results were seen when excluding BMI from the model.

In the path model without BMI, the significant adjusted variables are included as direct effects, with the effects of season of assessment and sex of the child being over twice those of paternal age and time spent in out of school sports clubs. In addition to these variables, significant indirect effects were seen of maternal age and Townsend socio-economic deprivation, both of which were mediated through the amount of time spent in out of school sports clubs (Figure 3a). In the path model including BMI, a number of additional variables were seen to have indirect effects mediated through contemporary BMI (Figure 3b). There were no significant interactions on any of the outcomes measures between sex and any of the potential predictor variables included in the analysis.

## Discussion

### Summary of Findings

In this study using objective measures of physical activity, we have shown sex and seasonal differences and an association with contemporary BMI for all three included outcome measures. Boys were more active and children were, on average, less active in the winter months. Increased BMI was associated with greater percentage of time spent in sedentary behaviour and with reduced total physical activity and percentage time spent in MVPA. Children of older fathers, as recorded at birth, had greater percentages of time spent in sedentary behaviour, while parental restrictions on time spent watching television was associated with decreased percentages of time spent in MVPA. Children who spent more time in out-of-school sports clubs had lower percentages of time spent in sedentary behaviour.

### Strengths and Limitations

The main strength of this study is the ability to analyse prospectively collected data from different stages of childhood simultaneously in relation to objective measures of physical activity and sedentary behaviour. This approach has been helpful in explaining health behaviours in other populations, but is novel in the context of the study of factors influencing childhood physical activity. Also, a path analysis of numerous factors allows both direct and indirect (i.e. mediating) effects to be estimated and their relative impacts on physical activity outcomes to be estimated. Paternal age was that recorded at the time of birth. As the biological or named father at birth may not necessarily be involved in with some of the children, there may be some uncertainties regarding the paternal age findings. Data on parental physical activity levels were not collected due to limited supplies of equipment.

The Actigraph GT1M is a uniaxial accelerometer (vertical), and has repeatedly been shown to measure physical activity and sedentary behaviour reliably in children. The lack of additional axes or the inclusion of a heart rate monitor to further validate the output are a limitation with the GT1M. However a great deal of data are now published with uniaxial accelerometers, allowing direct comparison of output from the GMS with other samples. The GMS has also used the GT1M at other timepoints, and when the children were younger, there were fewer comparative studies using alternative accelerometers published. Changing methods would also prevent us from tracking the children’s PA and SB reliably.

The use of path analysis has several benefits over standard linear regression, including a more illustrative quantification of the different pathways of influence. Nevertheless, some limitations require consideration. Firstly, the direction of each relationship has to be inferred by the researcher. This is generally less of an issue for longitudinal studies such as this, because the direction is often determined by clear temporal relationships. However, in this study it can be argued that the effects of BMI on the outcome measures could be bi-directional, hence the presentation of results with and without the BMI of the child. As with all forms of statistical modelling, path models are also sensitive to the specific features of the underlying data. It is therefore important to consider the characteristics of the cohort studied when estimating the relevance to other populations. Finally, path analysis is sensitive to error, since the standard deviation of each estimate strongly contributes to the final effect size. However, as the data for this study were collected prospectively, sources of error usually associated with retrospective data collection were minimised.

### Comparison with Previous Findings

Physical activity levels in this cohort were low, with the vast majority of children not reaching the 60 min/day MVPA guidelines [36]. This lack of physical activity is not restricted to children in this study, however, but is reported from diverse locations, including the ALSPAC cohort in southwest England [10] and the US Iowa Bone Development Study [11]. The data are therefore skewed towards inactivity. However, the number of significant associations shown with these data suggests that there remains enough variability and statistical power.

Sex differences in PA were also noted in the above studies, as well as the PEACHES study in London, UK [12] and the EarlyBird Study in Plymouth, UK [13]. There are no explanations for the differences in physical activity observed between boys and girls – but as similar observations have been made in 3–5year olds, the difference may be set early in life [37].

We found no associations between birth weight and any of the physical activity outcome variables. While this is consistent with a number of studies [7], [8], other studies have found associations with birth weight and there are suggestions that the association may be limited to very low birth weight individuals [9], rather than associations operating across the birth weight scale.

A recent review of after-school clubs to increase physical activity in children and adolescents [18] found mixed results, with some methodologically weak studies. It seems logical that children who choose to spend their leisure time taking part in sports clubs will increase their overall activity, and as children in the current study spent more time in outside-school sports clubs that may explain the lack of effect of school sports clubs. We have previously found a significant contribution of active commuting to school to overall physical activity and sedentary behaviour in this cohort of children [17], and a recent study of US adolescents found an inverse association between active commuting and BMI z-score [38] which highlights the importance of making physical activity an integral part of every child’s daily life. One limitation of the current study is that children self-reported sports club attendance, so could be prone to recall error, both over-and under-reporting the number/duration of clubs they attended.

Our previous paper on the correlates of physical activity in these children at a wave of data collection two years previously, while they were mean age 7 years [17] found an association of season with both physical actvity and sedentary behaviour, with less physical activity taking place in the winter months, an observation also reported in UK 11 y olds [15] and pre-school children [14]. This effect was still present in the current analyses at age 8–10 years. Cleland et al (2008) [39] observed a decrease in the amount of time children spent outdoors in the Australian cooler months compared with the warmer months, which may help to explain the season difference, particularly if sports clubs that take place outside are moved inside or cancelled. In addition, 10–11 year old UK children interviewed in focus groups reported rainy weather to be a barrier to their active outdoor play [40].

Television viewing/screen time (often used as a proxy for measurement of sedentary behaviour) has not been conclusively demonstrated to decrease time spent in physical activity, as the relationship between the two is complex, and probably act independently. We found that any level of television restriction was associated with decreased MVPA. Marshall et al (2004) [19] published a meta-analysis on media use, body fatness and physical activity in youth, and found very small displacement effects, mostly of vigorous intensity activity. However, most of the studies had not used an objective measurement of physical activity, but self- or parent-reported activity. Increased television viewing has been associated with an increase in body fat and reduced objectively-measured physical activity in preschool children (e.g. Jackson et al 2009 [35]) and with risk of overweight in 8 year olds [41]. However, as these studies measured body composition at only one time-point there is still the issue of direction of causality. Biddle et al., (2010) [21] reported that subjectively-measured sedentary behaviours such as TV viewing and electronic games show low-moderate tracking, and therefore interventions to reduce children’s screen time, despite having a small but statistically significant effect [42] may help to reduce risk of overweight in children and adolescents.

In conclusion, a range of factors, either directly or indirectly, influence physical activity in these children. However, associations differ by type of physical activity measurement. Exploring further the sex differences in physical activity would appear to be useful, as would encouraging children to join out of school sports clubs. This study has demonstrated that path analysis is a useful approach in understanding physical activity behaviour in children, and can provide insights not available from the methods used in more traditional studies of the correlates of children’s physical activity.
